# An Investigation of the Association between Health Screening and Dental Scaling in Korea

**DOI:** 10.3390/ijerph18084294

**Published:** 2021-04-18

**Authors:** Bo-Mi Shin, Jung-Sun Heo, Jae-In Ryu

**Affiliations:** 1 Department of Dental Hygiene, College of Dentistry, Gangneung Wonju National University, Gangneung-si 25457, Korea; purplebom@gwnu.ac.kr; 2 Department of Maxillofacial Biomedical Engineering and Institute of Oral Biology, College of Dentistry, Kyung Hee University, Seoul 02447, Korea; heojs@khu.ac.kr; 3 Department of Preventive and Social Dentistry, College of Dentistry, Kyung Hee University, Seoul 02447, Korea

**Keywords:** diagnostic screening programs, dental scaling, dental health service, health services accessibility

## Abstract

Dental disease is one of the most prevalent chronic diseases worldwide, and its expenditure is continuously increasing. Periodontal disease is increasing as a chronic non-communicable disease in adults and older people. Health screening has been shown to be cost-effective and improves the quality of life through the early detection of diseases. This study aimed to analyze the relationship between national health screening and dental scaling as a preventive service for periodontal disease. The study used sample cohort data from 2002 to 2015 provided by the National Health Insurance Sharing Service in South Korea. A logistic regression analysis of the utilization of dental scaling was performed to identify the independent effects of national health screening. People who underwent health screening showed a higher tendency to undergo dental scaling. Additionally, disparities in utilization according to socioeconomic status were reduced among those who underwent screening. The intervention to extend dental coverage could be more beneficial when combined with health screening, encouraging more people to participate and reducing inequalities in utilization.

## 1. Introduction

According to the Global Burden of Disease study, oral health has not improved over the past 25 years. The number of people with untreated oral diseases increased from 2.5 billion in 1990 to 3.5 billion in 2015 [1]. The direct treatment cost for oral diseases was estimated to be $290 billion [2], and dental expenditure accounts for the third highest proportion of health expenditures in the European Union [3]. In South Korea, the national dental expenditure amounted to KRW 4.6 trillion in 2019, an 18% increase compared to the previous year [4]. The relatively high rate of out-of-pocket payments, 57.5% for dental hospitals and 48.1% for dental clinics, within this expenditure, imposed an economic burden [5]. Periodontal disease is increasing as a chronic non-communicable disease in adults and older people [6,7,8]. Preventive and nonsurgical treatments are necessary before it develops as a severe disease [9,10]. Despite this need, barriers due to financial difficulties decrease the visibility of preventive services [11,12]. In this case, the reimbursement policy would be highly effective in eliminating this obstacle [13,14], affecting both the user and provider [15,16]. The percentage of people needing dental scaling was 66.3% in 2010 in Korea [17]. Since 2013, the government has implemented a coverage plan for annual dental scaling of the full mouth in adults. The National Health Insurance (NHI) covers healthcare services related to preventive treatment, diagnosis, treatment, rehabilitation, injuries, childbirth, and death [18]. The payment is based on a fee-for-service system that contributes to a third-party NHI corporation. Dental scaling service was permitted once a year as a supragingival approach to prevent periodontal disease and to treat early periodontitis [19,20].

After the policy, the number of people using dental scaling increased [21], and the number of people with healthy periodontal conditions increased as well [22]. However, there were socioeconomic inequalities [23], and some argued that it became severe after the policy that included dental scaling in the NHI [24]. With the implementation of this policy, the utilization rate increased in total, but the gap between socioeconomic groups increased. Universal access with universal dental coverage did not guarantee a decrease in the gap based on socioeconomic status. This might indicate that financial difficulty is not the only reason for not using dental care, especially preventive services. Diverse factors such as the patient’s socioeconomic status, educational level, and the way to provide intervention are crucial [25]. A pilot study was conducted in Korea, involving a registered dentist program. It provides preventive dental services to children by coordinating with compulsory annual oral examinations [26]. The participants showed a higher rate of using preventive dental services [27], and children from low-income groups experienced a decrease in the rate of dental visits for treatment [28,29]. This proved that preventive services could be effectively delivered after being combined with screening or examination in the whole or targeted population.

Health screening contributes to the early detection and treatment of diseases, thereby reducing the burden of medical expenditure and improving the quality of life and health status. National health screening was introduced in 1980 in South Korea and is now offered free of charge to all ages over one year of age. Health check-ups with oral examinations consist of health examinations, consultations, and education at nationally designated check-up institutions [30]. Recently, the World Health Organization suggested an extended concept of health examination that encompasses all steps of services in the process of linking health screening to disease management, including diagnosis and treatment [31]. South Korea also defined the purpose of health examination as the prevention and management of diseases following the Framework Act on Health Examinations. A follow-up system was introduced to encompass the scope of disease management. Extended health screening might increase accessibility and attenuate inequalities by combining with preventive services.

This study aimed to analyze the relationship between national health screening and dental scaling using the NHI data in South Korea.

## 2. Materials and Methods

### 2.1. Study Subjects

This study used sample cohort data from 2002 to 2015 provided by the National Health Insurance Sharing Service (NHISS) in Korea [32]. This cohort was the data standardized in a shareable form by extracting the sample to reduce limited access due to the large size and personally identifiable information issues. It allows the long-term observation of the same individuals as a cohort and connects qualification data, including socioeconomic variables (location of residence, month and date of death, cause of death, income rank, etc.) to treatment, medical check-up, and the clinic to enable the analysis of a causal or temporal relationship. This sample cohort included approximately one million qualified individuals extracted from Medicare recipients and NHI subscribers in 2006, which is 2% of the total population.

The study population included adults who were in the cohort between 2014 and 2015. Most insured patients receive medical check-ups every two years, but manual workers do this every year in Korea. Among the double records within two years, the first year was prioritized. Finally, the total number of study subjects was 851,792, with a single record per ID. This study was granted an exemption from the Institutional Review Board designated by the Ministry of Health and Welfare in South Korea (P01-201907-21-033).

### 2.2. Study Variable

Dental scaling was selected as the dependent variable in this study. The independent variables were sex, age, administrative districts, type of subscription, income quintile, disability, and national health screening. The type of subscription was classified as Medicare and health insurance, such as self-employed or employees. The income quantile was analyzed separately according to the type of subscription, because these had different contribution systems. Disability categorizes people with registration by social security. The subjects who received national health screening were the study group and those who did not were controls in this study.

### 2.3. Statistical Analysis

The utilization of dental scaling was examined using the chi-square test, according to independent variables. A logistic regression analysis of the utilization of dental scaling was performed to identify the independent effects of national health screening. The results are presented after separation by the study and control groups. The variables were included in a step-by-step analysis to evaluate the effects of the dependent variable. The study samples were analyzed as Model 1, adjusted for sex and age group; Model 2, adjusted for sex, age, and city level; Model 3, adjusted for sex, age, city, and disability; Model 4, adjusted for sex, age, city, disability, and insurance type or income quintile; and Model 5, adjusted for sex, age, city, disability, insurance type, income quintile, and health screening. The variables that were significant at a *p*-value < 0.05, were determined to significantly affect the dependent variable of dental scaling utilization. Data analysis was performed using the STATA version 15.1 statistical software package (StataCorp, College Station, TX, USA).

## 3. Results

There were 851,792 participants in the study cohort (Table 1). The proportion of male and female patients was similar in the study population. Slightly more than half of the participants lived in the province (53.9%). There were very few fragile people with disabilities or Medicaid (6.0% and 2.7%, respectively). Almost two-thirds of the participants were employees and their dependents. Both self-employed and employee groups had more people in higher-income quintiles. Half of the participants underwent general medical check-ups. A larger proportion of participants who were the youngest or the oldest, disabled, on Medicaid, or worse off did not undergo health screening (*p* < 0.001).

The utilization rate of dental scaling was 27.2% (Table 2). There were significant differences in all sociodemographic dependent variables for the use of this service (all *p* < 0.001). Participants who were female, younger, living in a metropolitan city, without disability, or an employee, had a higher income, or underwent national health screening showed a higher opportunity to experience dental scaling.

Significant differences in dental scaling by national health screening were observed even after adjusting for all the sociodemographic variables in Table 3, according to the logistic regression model (*p* < 0.001). The experience of screening increased the chance of dental scaling by as much as 1.63 times compared to people who did not. The youngest people showed a four-fold higher tendency to undergo dental scaling than the older ones. The self-employed and employees had a difference compared to the Medicaid beneficiaries, with a 1.36 to 1.70 times higher chance of experiencing the treatment. Women, dwellers in the metropolitan city area, or people without disabilities had a higher chance of receiving dental scaling in the fully adjusted model.

Table 4 separates the participants into groups based on their experience with the national health screening program and insurance type. The gaps were larger in the group without health screening, based on all independent variables. In particular, dental scaling ratios were the highest in the youngest group without health screening, even in a fully adjusted model. The scale of inequality by income quintile was larger in the self-employed group, and the richest group showed a 1.91 times higher chance of dental scaling than the poorest group. It was lower in the employee group, and the pattern was U-shaped with a long end in the higher quintiles.

## 4. Discussion

This study showed that the likelihood of dental scaling was higher in the group of people who participated in the national health screening program. The gap in sociodemographic information was larger in the group of people without a health screening.

Health screening is a critical factor in dental scaling. Health screening is a service that prevents disease severity through early diagnosis. A 10-year follow-up of the United Kingdom Multicenter Aneurysm Screening Study (MASS) investigated the effects of abdominal aortic aneurysm ultrasound screening on mortality, demonstrating that the mortality was decreased in the group invited for screening. The screening resulted in a 45% relative risk reduction in this study [33]. Studies that have examined the effects of health screening and used simulation models to evaluate the economic feasibility have also shown cost-effectiveness [34], and support that it improves the quality of life through the early detection of diseases [35]. This improvement might be due to a higher tendency to make healthier decisions among people who received the screening. Moreover, the diagnosis could lead to a healthier behavior [36,37,38,39]. Medical or dental professionals examine the patients and explain the diagnosis, playing an important role in encouraging patients to have the necessary treatment and to make healthier choices [40,41]. Further, medical professionals can do this for dental care by recommending proper dental services for children and their families [42]. The diagnosis would promote patient participation, and suggestions from professionals can encourage them to seek preventive services.

The disparities in the utilization of dental scaling according to demographics were more severe in those who did not undergo health screening. A British study also revealed that education and social hierarchy were significantly associated with the tendency to use treatments or preventive dental services, even in countries with equitable access [43,44,45]. Disability is another barrier to accessibility for dental care in children [46,47] and adults [48,49]. Sex showed a significant relationship with the utilization of dental services; females had a higher tendency to use dental services than males [50,51,52,53]. Rural areas with a lack of service utility and personnel also have access limitations [54,55,56,57,58,59,60]. However, interventions for children with registered dentist programs did not show significant inequalities based on these factors [61]. This project provided preventive dental services under compulsory oral examinations, in which all the students were required to visit dental clinics. The participants showed a higher rate of use of preventive dental services, and the gap by income quintiles disappeared. Another project, the Well-Integrated Screening and Evaluation for Women Across the Nation, WISEWOMAN, was implemented to target low-income, under, or uninsured women [62]. Middle-aged participants underwent screening to reduce the risk of heart disease and an intervention to eat healthy, increase physical activity, and quit smoking. Many researchers have shown that it causes behavioral changes and clinical improvement [63,64,65,66,67,68,69,70,71]. Interventions might be necessary to connect with screening, in which people can identify their status and be motivated to use preventive services from the advice of reliable professionals.

There was a larger age gap in people without a health screening. The youngest group had a five-fold higher probability of using dental scaling. Similar results have been reported in other studies of the rate of dental scaling [23,72,73]. Even after dental insurance with a coverage extension that induced a change in utilization, the absolute rate decreased with age [21,74]. A study from England showed that health check attendees decreased with age in both the underserved and general populations [75]. In addition, the utilization ratio was lower in the Taiwanese elderly with disabilities, where dental scaling services were free of charge every six months [76]. This might be related to the edentulous condition, which was 8.7% among adults over 70 years in Korea [77]. However, the average number of natural teeth was 16.9, indicating that dental scaling is still needed. Rada et al. analyzed the utilization of dental services with sociodemographic factors through a systematic review [78]. They concluded that the odds ratio of dental-service utilization differed according to the country profile in the elderly group. For example, North America or high-income Asia had a lower odds ratio, but Southeast Asia showed higher ratios, while no significant difference was observed in other countries. A Japanese study revealed that age did not significantly affect the dental utilization of adults [79]. The differences might be due to the system and coverage of dental care by the country and the dissimilar background with cultural literacy by generation. Further studies are needed to determine the relationship between the utilization of preventive dental services and age or generation, and the reason for this tendency.

This study has several limitations. First, health screening experience was used as a variable to categorize participants with dental scaling instead of an oral examination. This study used a sample cohort database that included only health screening records. General health screening by the National Health Insurance Service in Korea included health checkups, oral check-ups, cancer screenings, and infant health screenings. However, a sample cohort DB provided a record of general health screening only; therefore, it was impossible to use the experience variable for an oral examination. There is another dataset, the medical check-up cohort DB, which included approximately half a million NHI subscribers who received general medical check-ups and were aged 40 to 79 years between 2002 and 2003. Further studies are needed to apply this dataset focusing on the association between oral examination and preventive dental services, even with the restriction of age groups. Second, health behaviors related to the utilization of dental services were not included in this study. Andersen’s behavioral model of service utilization explained that there are several domains, such as predisposing, enabling, need, and behavioral factors, which result in outcomes of perceived health, evaluated health, or patient satisfaction [80,81,82,83]. As mentioned above, the dataset provided by the NHISS had a limitation on the range of variables related to oral health. Further studies are needed to determine the relationship between these health behaviors and other factors. Third, all the tables show the results for the whole population, not separated by sex. This study focused on the relationship between health screening and dental scaling. Sex could be a compounding variable closely related to these factors, for example, the utilization of dental services. This study showed slight differences by sex, but it was relatively small compared to the other factors. Recently, the researchers showed the results separately by sex; therefore, these results need to be considered.

## 5. Conclusions

People who had experience in health screening showed a higher tendency to use dental scaling. In addition, disparities in utilization according to socioeconomic status were reduced in the group of people who underwent screening. The intervention to extend dental coverage could be more beneficial when combined with health screening, which encourages more people to participate and reduces inequalities in utilization.

## Figures and Tables

**Table 1:** The distribution of study sample between 2014 and 2015 in the National Health Insurance Sharing Service (NHISS).

Variables	Subject		Health Screening		No Health Screening		
	N	(%)	N	(%)	N	(%)	
Total	851,792	(100.0)	432,354	(50.8) (100.0)	419,428	(49.2) (100.0)	
Gender							
Male	422,434	(49.6)	219,726	(50.8)	202,708	(48.3)	
Female	429,358	(50.4)	212,638	(49.2)	216,720	(51.7)	
Age							
70+	94,570	(11.1)	43,898	(10.2)	50,672	(12.1)	***
60–69	97,657	(11.5)	66,627	(15.4)	31,030	(7.4)	
50–59	167,534	(19.7)	105,375	(24.4)	62,159	(14.8)	
40–49	181,963	(21.4)	108,314	(25.1)	73,649	(17.6)	
30–39	159,818	(18.8)	70,547	(16.3)	89,271	(21.3)	
20–29	150,250	(17.6)	37,603	(8.7)	112,647	(26.9)	
City							
Province	458,866	(53.9)	236,785	(54.8)	222,081	(52.9)	
Metropolitan city	392,926	(46.1)	195,579	(45.2)	197,347	(47.1)	
Disability							
Yes	50,967	(6.0)	25,164	(5.8)	25,803	(6.2)	***
No	800,825	(94.0)	407,200	(94.2)	393,625	(93.8)	
Insurance type							
Medicaid	23,424	(2.7)	5547	(1.3)	17,877	(4.3)	***
Self-employed	254,410	(29.9)	95,197	(22.0)	159,213	(38.0)	
Employee	573,958	(67.4)	331,620	(76.7)	242,338	(57.8)	
Income quintile							
Self-employed							
1st	31,188	(12.3)	9753	(10.2)	21,435	(13.5)	***
2nd	37,592	(14.8)	11,890	(12.5)	25,702	(16.1)	
3rd	51,914	(20.4)	18,412	(19.3)	33,502	(21.0)	
4th	62,287	(24.5)	24,803	(26.1)	37,484	(23.5)	
5th	71,418	(28.1)	30,337	(31.9)	41,081	(25.8)	
Employee							
1st	94,728	(16.7)	50,853	(15.6)	43,875	(18.3)	***
2nd	94,938	(16.8)	54,412	(16.7)	40,526	(16.9)	
3rd	102,780	(18.1)	61,870	(19.0)	40,910	(17.1)	
4th	120,256	(21.2)	74,626	(22.9)	45,630	(19.0)	
5th	153,665	(27.1)	84,669	(25.9)	68,996	(28.8)	

*** *p* < 0.001.

**Table 2:** The distribution of study sample with scaling between 2014 and 2015 in the National Health Insurance Sharing Service (NHISS).

Variables	Subject			Health Screening			No Health Screening		
	N	(%)		N	(%)		N	(%)	
Total	231,557	(27.2)	***	136,438	(31.6)		95,119	(22.7)	***
Gender									
Male	110,464	(26.1)	***	68,108	(31.0)	***	42,356	(20.9)	***
Female	121,093	(28.2)		68,330	(32.1)		52,763	(24.3)	
Age									
70+	8982	(9.5)	***	5890	(13.4)	***	3092	(6.1)	***
60–69	23,739	(24.3)		18,281	(27.4)		5458	(17.6)	
50–59	47,740	(28.5)		34,658	(32.9)		13,082	(21.0)	
40–49	53,763	(29.5)		37,951	(35.0)		15,812	(21.5)	
30–39	51,300	(32.1)		25,848	(36.6)		25,452	(28.5)	
20–29	46,033	(30.6)		13,810	(36.7)		32,223	(28.6)	
City									
Province	117,238	(25.5)	***	70,134	(29.6)	***	47,104	(21.2)	***
Metropolitan city	114,319	(29.1)		66,304	(33.9)		48,015	(24.3)	
Disability									
Yes	8530	(16.7)	***	5522	(21.9)	***	3008	(11.7)	***
No	223,027	(27.8)		130,916	(32.2)		92,111	(23.4)	
Insurance type									
Medicaid	3194	(13.6)	***	1295	(23.3)	***	1899	(10.6)	***
Self-employed	58,636	(23.0)		26,345	(27.7)		32,291	(20.3)	
Employee	169,727	(29.6)		108,798	(32.8)		60,929	(25.1)	
Income quintile									
Self-employed									
1st	5087	(16.3)	***	1989	(20.4)	***	3098	(14.5)	***
2nd	7235	(19.2)		2845	(23.9)		4390	(17.1)	
3rd	10,915	(21.0)		4654	(25.3)		6261	(18.7)	
4th	14,795	(23.8)		6790	(27.4)		8005	(21.4)	
5th	20,604	(28.8)		10,067	(33.2)		10,537	(25.6)	
Employee									
1st	26,255	(27.7)	***	15,409	(30.3)	***	10,846	(24.7)	***
2nd	25,914	(27.3)		16,568	(30.4)		9346	(23.1)	
3rd	29,543	(28.7)		19,563	(31.6)		9980	(24.4)	
4th	36,180	(30.1)		24,903	(33.4)		11,277	(24.7)	
5th	49,661	(32.3)		30,725	(36.3)		18,936	(27.4)	

*** *p* < 0.001.

**Table 3:** Odds ratio (OR) and 95% confidence interval (CI) estimated from logistic regression models for dental scaling in total in the NHISS (N = 851,791).

(=Reference)	Unadjusted	Model 1	Model 2	Model 3	Model 4	Model 5
Gender (=Male)						
Female	1.11 (1.10–1.12) ***	1.17 (1.16–1.18) ***	1.17 (1.04–1.07) ***	1.16 (1.15–1.17) ***	1.16 (1.15–1.17) ***	1.18 (1.17–1.19) ***
Age (=70+)						
60–69	3.06 (2.98–3.14) ***	3.11 (3.03–3.19) ***	3.08 (3.00–3.16) ***	3.02 (2.94–3.10) ***	3.00 (2.92–3.08) ***	2.74 (2.67–2.81) ***
50–59	3.80 (3.71–3.89) ***	3.87 (3.78–3.96) ***	3.83 (3.74–3.93) ***	3.71 (3.62–3.80) ***	3.73 (3.64–3.82) ***	3.48 (3.40–3.57) ***
40–49	4.00 (3.90–4.09) ***	4.08 (3.98–4.17) ***	4.05 (3.95–4.14) ***	3.88 (3.78–3.97) ***	3.88 (3.79–3.98) ***	3.69 (3.61–3.79) ***
30–39	4.50 (4.40–4.61) ***	4.60 (4.49–4.71) ***	4.55 (4.44–4.66) ***	4.34 (4.23–4.44) ***	4.20 (4.10–4.30) ***	4.37 (4.26–4.48) ***
20–29	4.21 (4.11–4.31) ***	4.31 (4.20–4.41) ***	4.26 (4.16–4.37) ***	4.05 (3.95–4.15) ***	3.94 (3.85–4.04) ***	4.51 (4.40–4.62) ***
City (=Province)						
Metropolitan city	1.20 (1.18–1.21) ***		1.17 (1.15–1.18) ***	1.16 (1.15–1.17) ***	1.16 (1.15–1.17) ***	1.17 (1.16–1.19) ***
Disability (=Yes)						
No	1.92 (1.88–1.97) ***			1.41 (1.38–1.45) ***	1.31 (1.27–1.34) ***	1.28 (1.25–1.32) ***
Insurance type (=Medicaid)						
Self-employed	1.90 (1.83–1.97) ***				1.40 (1.35–1.46) ***	1.36 (1.31–1.42) ***
Employee	2.66 (2.56–2.76) ***				1.97 (1.89–2.05) ***	1.70 (1.63–1.77) ***
Health screening (=No)						
Yes	1.57 (1.56–1.59) ***					1.63 (1.61–1.64) ***

*** *p* < 0.001. Model 1: adjusted for gender and age; Model 2: adjusted for gender, age, and city type; Model 3: adjusted for gender, age, city, and disability; Model 4: adjusted for gender, age, city, disability, and insurance type; Model 5: adjusted for gender, age, city, disability, insurance type, and national health screening.

**Table 4:** Odds ratio (OR) and 95% confidence interval (CI) estimated from logistic regression models for dental scaling by insurance types in the NHISS.

	Unadjusted		Model 4	
(=Reference)	With Health Screening	Without Health Screening	With Health Screening	Without Health Screening
Total				
Gender (= Male)				
Female	1.05 (1.04–1.07) ***	1.22 (1.20–1.24) ***	1.10 (1.09–1.11) ***	1.27 (1.25–1.29) ***
Age (=70+)				
60–69	2.44 (2.36–2.52) ***	3.28 (3.13–3.44) ***	2.41 (2.34–2.49) ***	3.30 (3.15–3.46) ***
50–59	3.16 (3.07–3.26) ***	4.10 (3.94–4.27) ***	3.11 (3.01–3.20) ***	4.14 (3.97–4.31) ***
40–49	3.48 (3.38–3.59) ***	4.21 (4.04–4.38) ***	3.38 (3.28–3.49) ***	4.20 (4.03–4.37) ***
30–39	3.73 (3.62–3.85) ***	6.14 (5.90–6.38) ***	3.54 (3.43–3.65) ***	5.74 (5.52–5.97) ***
20–29	3.75 (3.62–3.88) ***	6.17 (5.93–6.41) ***	3.51 (3.39–3.64) ***	5.84 (5.61–6.07) ***
City (=Province)				
Metropolitan city	1.22 (1.20–1.23) ***	1.19 (1.18–1.21) ***	1.19 (1.17–1.20) ***	1.16 (1.14–1.17) ***
Disability (=Yes)				
No	1.69 (1.63–1.74) ***	2.32 (2.23–2.41) ***	1.25 (1.21–1.29) ***	1.34 (1.29–1.40) ***
Insurance type (=Medicaid)				
Self-employed	1.26 (1.18–1.34) ***	2.14 (2.04–2.25) ***	1.30 (1.22–1.39) ***	1.32 (1.25–1.39) ***
Employee	1.60 (1.51–1.71) ***	2.83 (2.69–2.97) ***	1.56 (1.46–1.66) ***	1.72 (1.63–1.81) ***
Self-employed				
Gender (=Male)				
Female	1.10 (1.07–1.14) ***	1.29 (1.26–1.32) ***	1.12 (1.09–1.15) ***	1.33 (1.30–1.36) ***
Age (=70+)				
60–69	2.36 (2.22–2.51) ***	2.87 (2.64–3.13) ***	2.22 (2.08–2.35) ***	2.85 (2.62–3.10) ***
50–59	2.73 (2.57–2.89) ***	3.19 (2.96–3.43) ***	2.60 (2.45–2.75) ***	3.25 (3.02–3.51) ***
40–49	2.96 (2.80–3.14) ***	3.20 (2.97–3.44) ***	2.90 (2.73–3.08) ***	3.30 (3.07–3.56) ***
30–39	2.65 (2.45–2.85) ***	4.86 (4.52–5.23) ***	2.79 (2.59–3.01) ***	5.01 (4.66–5.40) ***
20–29	2.44 (2.23–2.67) ***	4.95 (4.61–5.32) ***	2.49 (2.27–2.72) ***	4.94 (4.59–5.32) ***
City (=Province)				
Metropolitan city	1.23 (1.20–1.27) ***	1.15 (1.13–1.18) ***	1.19 (1.16–1.22) ***	1.12 (1.09–1.15) ***
Disability (=Yes)				
No	1.50 (1.41–1.60) ***	1.79 (1.67–1.91) ***	1.18 (1.11–1.26) ***	1.17 (1.09–1.26) ***
Income quintile (=1st)				
2nd	1.23 (1.15–1.31) ***	1.22 (1.16–1.28) ***	1.10 (1.03–1.17) **	1.10 (1.05–1.16) ***
3rd	1.32 (1.24–1.40) ***	1.36 (1.30–1.43) ***	1.19 (1.12–1.26) ***	1.22 (1.17–1.28) ***
4th	1.47 (1.39–1.56) ***	1.61 (1.54–1.68) ***	1.32 (1.24–1.40) ***	1.46 (1.39–1.52) ***
5th	1.94 (1.84–2.05) ***	2.04 (1.95–2.13) ***	1.80 (1.71–1.91) ***	1.91 (1.83–2.00) ***
Employee				
Gender (=Male)				
Female	1.05 (1.04–1.07) ***	1.18 (1.16–1.20) ***	1.15 (1.13–1.17) ***	1.21 (1.19–1.23) ***
Age (=70+)				
60–69	2.50 (2.41–2.60) ***	3.47 (3.27–3.69) ***	2.72 (2.61–2.83) ***	3.73 (3.51–3.96) ***
50–59	3.39 (3.27–3.51) ***	4.78 (4.53–5.04) ***	3.60 (3.47–3.74) ***	5.10 (4.83–5.39) ***
40–49	3.68 (3.55–3.81) ***	5.01 (4.75–5.28) ***	3.82 (3.69–3.96) ***	5.16 (4.89–5.44) ***
30–39	3.88 (3.74–4.02) ***	6.52 (6.20–6.85) ***	4.27 (4.12–4.44) ***	6.82 (6.48–7.18) ***
20–29	3.94 (3.79–4.10) ***	6.35 (6.04–6.67) ***	4.56 (4.37–4.75) ***	6.66 (6.33–7.01) ***
City (=Province)				
Metropolitan city	1.21 (1.93–1.23) ***	1.20 (1.18–1.22) ***	1.18 (1.16–1.20) ***	1.16 (1.14–1.18) ***
Disability (=Yes)				
No	1.70 (1.64–1.76) ***	2.51 (2.38–2.65) ***	1.20 (1.16–1.25) ***	1.38 (1.31–1.47) ***
Income quintile (=1st)				
2nd	1.01 (1.98–1.03) ***	0.91 (0.88–0.94) ***	0.95 (0.93–0.98) ***	0.91 (0.88–0.94) ***
3rd	1.06 (1.04–1.09)	0.98 (0.95–1.01)	1.01 (0.98–1.04)	1.00 (0.97–1.03)
4th	1.15 (1.12–1.18) ***	1.00 (0.97–1.03)	1.16 (1.13–1.19) ***	1.08 (1.05–1.11) ***
5th	1.31 (1.28–1.34) ***	1.15 (1.12–1.18) ***	1.48 (1.45–1.52) ***	1.37 (1.33–1.41) ***

** *p* < 0.01, *** *p* < 0.001. Model 4: adjusted for gender, age, city, disability, and insurance type or income quintile.

## Data Availability

Data is available from NHIS upon request.
